# Trends in prevalence of extended-spectrum beta-lactamase-producing *Escherichia coli* isolated from patients with community- and healthcare-associated bacteriuria: results from 2014 to 2020 in an urban safety-net healthcare system

**DOI:** 10.1186/s13756-021-00983-y

**Published:** 2021-08-11

**Authors:** Eva Raphael, M. Maria Glymour, Henry F. Chambers

**Affiliations:** 1 Department of Epidemiology and Biostatistics , University of California, San Francisco, San Francisco, CA USA; 2 Department of Family and Community Medicine , University of California, San Francisco, San Francisco, CA USA; 3 Department of Medicine, University of California, San Francisco, San Francisco, CA USA; 4 Zuckerberg San Francisco General Hospital, 995 Potrero Avenue, Ward 83, San Francisco, CA 94110 USA; 5 Zuckerberg San Francisco General Hospital, 1001 Potrero Avenue, San Francisco, CA 94110 USA

**Keywords:** Extended-spectrum beta-lactamase, *Escherichia coli*, Antimicrobial resistance, Bacteriuria, Risk factors

## Abstract

**Background:**

The prevalence of infections caused by extended-spectrum beta-lactamase producing *Escherichia coli* (ESBL-*E. coli*) is increasing worldwide, but the setting in which this increase is occurring is not well defined. We compared trends and risk factors for ESBL-*E. coli* bacteriuria in community vs healthcare settings.

**Methods:**

We collected electronic health record data on all patients with *E. coli* isolated from urine cultures in a safety-net public healthcare system from January 2014 to March 2020. All analyses were stratified by healthcare-onset/associated (bacteriuria diagnosed > 48 h after hospital admission or in an individual hospitalized in the past 90 days or in a skilled nursing facility resident, N = 1277) or community-onset bacteriuria (bacteriuria diagnosed < 48 h after hospital admission or in an individual seen in outpatient clinical settings without a hospitalization in the past 90 days, N = 7751). We estimated marginal trends from logistic regressions to evaluate annual change in prevalence of ESBL-*E. coli* bacteriuria among all bacteriuria. We evaluated risk factors using logistic regression models.

**Results:**

ESBL-*E. coli* prevalence increased in both community-onset (0.91% per year, 95% CI 0.56%, 1.26%) and healthcare-onset/associated (2.31% per year, CI 1.01%, 3.62%) bacteriuria. In multivariate analyses, age > 65 (RR 1.88, CI 1.17, 3.05), male gender (RR 2.12, CI 1.65, 2.73), and Latinx race/ethnicity (RR 1.52, CI 0.99, 2.33) were associated with community-onset ESBL-*E. coli*. Only male gender (RR 1.53, CI 1.03, 2.26) was associated with healthcare-onset/associated ESBL-*E. coli*.

**Conclusions:**

ESBL-*E. coli* bacteriuria frequency increased at a faster rate in healthcare-associated settings than in the community between 2014 and 2020. Male gender was associated with ESBL-*E. coli* bacteriuria in both settings, but additional risks—age > 65 and Latinx race/ethnicity—were observed only in the community.

**Supplementary Information:**

The online version contains supplementary material available at 10.1186/s13756-021-00983-y.

## Keypoints

The frequency of bacteriuria caused by *Escherichia coli* producing extended-spectrum beta-lactamase (ESBL) increased from 2014 to 2020 in community and healthcare settings among patients diagnosed at a public healthcare system. Risk factors differed between community-onset versus healthcare-onset/associated ESBL-*E. coli* bacteriuria.

## Introduction

Infections caused by extended-spectrum beta-lactamase (ESBL)-producing Enterobacteriaceae are a growing public health threat [1, 2]. In 2019, the Center for Disease Control and Prevention (CDC) designated ESBL-producing Enterobacteriaceae as”serious threat” pathogens [2]. While the first US cases of infections caused by ESBL-producing *Escherichia coli* (ESBL-*E. coli*) were identified in a skilled nursing facility, it is now a nascent concern in community settings [3–8]. Recent hospitalization and prior antibiotic use are major drivers of ESBL-*E. coli* infections, but factors unrelated to healthcare are increasingly recognized, including international travel and consumption of meat contaminated with ESBL-*E. coli*.[9–14] However, most studies compare infection with ESBL-*E. coli* to infection with drug-susceptible *E. coli* to assess risk for ESBL-*E. coli* infections [11]. As such, identified risks may determine infection with antimicrobial resistant *E. coli* and not necessarily ESBL-*E. coli*. Furthermore, despite the increasing prevalence of community-onset ESBL-*E. coli* infections*,* little is known about risk factors for such infections in community settings.

Previously, we found increasing trends in ESBL-*E. coli* bacteriuria in the San Francisco safety-net public healthcare system from 2012 to 2018, but community-onset cases could not be differentiated from those associated with healthcare exposure [15]. Here, we compared community-onset vs healthcare-onset/associated bacteriuria episodes caused by ESBL-*E. coli* in the same public healthcare system from 2014 to 2020. This healthcare system serves a multiethnic, low-income, under-studied population residing in various neighborhoods. Identifying risk factors specific to community-onset bacteriuria caused by ESBL-*E. coli* in this population is paramount to devising effective antibiotic stewardship efforts and targeted interventions to reduce transmission within communities.

## Methods

### Study design and settings

This is an observational study drawn from electronic medical record (eMR) data at the San Francisco public healthcare system for all patients whose urine culture grew *E. coli* from January 2014 to March 2020. This healthcare system includes 15 primary care outpatient clinics as part of the San Francisco Health Network (SFHN), the San Francisco General Hospital (SFGH), an acute care hospital, and Laguna Honda Hospital (LHH), a skilled nursing facility. SFHN patients and LHH residents are usually hospitalized at SFGH. The SFGH microbiology laboratory processes all laboratory tests for this public healthcare system. Data on all urine cultures were collected, including bacterial species and antimicrobial susceptibility test results. We analyzed bacteriuria episodes, which may represent either urinary tract infection or asymptomatic bacteriuria, caused by *E. coli*. Bacteriuria episodes were defined as a single urine culture growing E. coli. If multiple cultures were sent on the same day, only one culture was considered. *E. coli*-positive urine cultures from separate days were considered to be separate episodes, as we could not differentiate between an untreated and a repeat urinary tract infection.

### Exposure and outcome measures

To evaluate prevalence trends, we defined culture date as years since baseline (January 2014). Our primary independent variables were extracted from eMRs: age at time of culture (0–17, 18–34, 35–64, or over 65 years); gender (male or female); race/ethnicity (Asian, Black, Latinx, White, or Other); and preferred language (any Chinese dialect, English, Spanish, or Other).

Healthcare-onset/associated bacteriuria episodes were defined as cases in which a urine culture, obtained from a) inpatients after at least 48 h of hospital admission, b) outpatients who had been hospitalized in the 90 days prior to culture, or c) residents of the skilled nursing facility, grew *E. coli*. Community-onset *E. coli* bacteriuria episodes were defined as cases in which a urine culture, obtained in a) an outpatient clinic or emergency department setting, or b) within 48 h of inpatient admission, grew *E. coli*. These simplified definitions for healthcare onset/associated and community-onset were created due to the small sample size of community-onset healthcare-associated bacteriuria episodes. We sought to identify factors associated with antimicrobial resistance in community-onset bacteriuria other than prior hospitalization, which is an already well-studied risk factor for antimicrobial resistance. Here, we assumed that individuals who had been hospitalized in the 90 days prior to urine culture would have similar healthcare exposures to those individuals whose urine cultures were obtained after 48 h of hospitalization.

### Antimicrobial susceptibility testing (AST)

The microbiology laboratory performs AST with Microscan and disk diffusion tests, with reports of resistance based on CLSI breakpoint standards [16]. The microbiology laboratory reports extended-spectrum beta-lactamase producing *E. coli* (ESBL-*E. coli*) as an *E. coli* strain resistant to ceftazidime or cefotaxime and inhibited by clavulanic acid using broth microdilution, per 2016 CLSI guidelines [16]. Results reported as “intermediate resistance” were considered resistant in this study (“Appendix”).

Bacteriuria episode caused by ESBL-*E. coli* was the main outcome of interest. Sub-analyses included *E. coli* with resistance to nitrofurantoin, trimethoprim-sulfamethoxazole, ciprofloxacin (most commonly used to treat urinary tract infections), any resistance (defined as resistance to any antibiotic class based on all antibiotics tested routinely in the microbiology laboratory, see “Appendix” for a complete list), and multidrug resistance (defined as resistance to 3 or more classes of antibiotics). Sub-analyses initially included resistance to aminoglycosides and carbapenems; Additional file 1: Table S7 reports percentage resistance per year for each bacteriuria type and place of care.

### Statistical data analysis

Descriptive statistics, including frequencies and percentages for categorical data and mean values with standard deviations for continuous data, were used to summarize key exposure and outcome variables. Differences in overall frequency of ESBL-*E. coli* and patient characteristics for community-onset versus healthcare-onset/associated bacteriuria were evaluated with chi-squared tests and t-tests. Annual changes in resistance to antimicrobial agents from 2014 to 2020 were fit with logistic regression models and trends were estimated based on marginal effects, separately for community-onset and for healthcare-onset/associated bacteriuria. To assess changes in the overall patient population, annual change in racial/ethnic distribution of patients with *E. coli* bacteriuria were also fit with logistic regression models and trends were estimated based on marginal effects. Unadjusted and covariate adjusted logistic regression models were performed separately for community-onset bacteriuria and healthcare-onset/associated bacteriuria to assess which demographic characteristics of patients with bacteriuria predicted *E. coli* resistant to antimicrobial agents (considering as separate outcomes: ESBL-*E. coli;* resistance to each of 3 antibiotics separately [nitrofurantoin, trimethoprim-sulfamethoxazole, and ciprofloxacin]; any drug resistance; and multidrug resistance). Bootstrap (clustered at the individual level) confidence intervals were reported for adjusted logistic regressions to adjust for repeated measures on unique patients [17]. All analyses were conducted by RStudio4 version 1.3.1073. We report 95% confidence intervals to characterize uncertainty in our effect estimates. Sub-analyses were also performed separately for place of care: outpatient, inpatient, and skilled nursing facility.

## Results

### Characteristics of the study samples and patients

From January 2014 to March 2020, 82,800 urine samples were processed at the SFGH clinical microbiology laboratory. Of these, 13,522 urine cultures grew an identifiable organism, of which 9028 (67%) grew *E. coli* (7751 community-onset and 1277 healthcare-onset/associated). Of the 9028 urine cultures, 224 (2%) were obtained from catheterizations and 8804 (98%) from clean catch urine collections.

We identified 6291 unique patients with an *E. coli* bacteriuria episode. There were 5576 patients who met the definition of a community-onset *E. coli* bacteriuria. In addition, 926 patients had a healthcare-onset/associated bacteriuria; given that a patient may have had multiple bacteriuria episodes, these were not mutually exclusive (Table 1). There were 4844 outpatients, 1183 inpatients, and 264 skilled nursing facility residents. Fifteen hundred patients had more than one *E. coli* bacteriuria episode; one patient had as many as 27 episodes. Patients with community-onset bacteriuria (mean age 46) were younger compared to patients with healthcare-onset/associated bacteriuria (mean age 60). Patients with bacteriuria were predominantly women (85%). The study population was multiethnic, with 40% Latinx, 20% Asian, 18% White, and 14% Black patients. Demographic characteristics of outpatients, inpatients and skilled nursing facility residents can be found in Supplemental Table 1. We conducted analyses to assess change in percent race/ethnicity distribution in patients with *E. coli* bacteriuria from 2014 to 2020. We found that percent annual change in Latinx patients with *E. coli* bacteriuria increased by 1.44% (95% CI 0.78, 2.10), whereas percent change in Black patients (− 0.5%; 95% CI − 0.99, − 0.04) and patients identified as other (− 0.76%; 95% CI − 1.12, − 0.41) decreased annually. Annual percent change in other race/ethnic groups were not significant (data not shown).

**Table 1:** Demographic characteristics of patients with community-onset vs healthcare-onset/associated *E. coli* bacteriuria episodes, San Francisco, 2014–2020

	Number of patients, N (%)		
	Community-onset N = 5576	Healthcare-onset/associated N = 926	Total N = 6291
**Age category (years)**			
0–17	329 (6)	12 (1)	334 (5)
18–34	1619 (29)	105 (11)	1677 (27)
35–64	2483 (45)	407 (44)	2786 (44)
65 +	1145 (21)	402 (43)	1494 (24)
**Gender**			
Female	4841 (87)	639 (69)	5320 (85)
Male	731 (13)	287 (31)	967 (15)
Unknown	4 (< 1)		4 (< 1)
**Race/ethnicity**			
Asian	1079 (19)	179 (19)	1231 (20)
Black	746 (13)	175 (19)	896 (14)
Latinx	2335 (42)	254 (27)	2494 (40)
Other	408 (7)	64 (7)	452 (7)
Unknown/Declined	90 (2)	8 (1)	97 (2)
White	918 (16)	246 (27)	1121 (18)
**Preferred language**			
Chinese dialect	426 (8)	90 (10)	505 (8)
English	3167 (57)	626 (68)	3664 (58)
Unknown	82 (1)	15 (2)	98 (2)
Other	227 (4)	37 (4)	256 (4)
Spanish	1674 (30)	155 (17)	1768 (28)

Data from a public healthcare system including inpatient and outpatient services and a skilled nursing facility

### Prevalence of antimicrobial resistant E. coli

Table 2 shows the overall frequency at which the *E. coli* bacteriuria episodes were resistant to antimicrobial agents. ESBL-*E. coli* bacteriuria frequency was higher in healthcare onset/associated bacteriuria episodes (24%) compared to those of community-onset bacteriuria (8%) (*P* < 0.001) (Tables 2 and 3). ESBL-*E. coli* accounted for only 13% of community-onset but 34% of healthcare onset/associated antimicrobial resistant *E. coli* bacteriuria episodes (*P* < 0.001). Bacteriuria episodes caused by ESBL-*E. coli* had 4.14 (95% confidence interval [CI] 3.41, 5.02) times the risk of nitrofurantoin resistance, 3.89 (95% CI 3.41, 4.43) times the risk of trimethoprim-sulfamethoxazole resistance, and 12.95 (95% CI 11.15, 15.05) times the risk of ciprofloxacin resistance compared to bacteriuria caused by non-ESBL-*E. coli*. Amongst 14 carbapenem-resistant *E. coli* bacteriuria episodes, 6 were identified as community-onset, of which 2 were caused by non-ESBL-*E. coli*. All eight carbapenem-resistant *E. coli* strains causing healthcare onset/associated bacteriuria were ESBL-*E. coli*. The majority of ESBL-*E. coli* isolates were multidrug resistant (72% of 931) but only a minority (39% of 1707 isolates) of multidrug resistant episodes were due to ESBL-*E. coli*.

**Table 2:** Overall frequency of antimicrobial resistant *E. coli* from community-onset vs healthcare-onset/associated bacteriuria episodes, 2014–2020

	Number of antimicrobial resistant *E. coli* bacteriuria episodes, n (%)					
	NIT	T/S	CIP	ESBL	Any AMR	MDR
Community onset*	129 (2)	2691 (35)	1537 (20)	623 (8)	4719 (61)	1306 (16.85)
Healthcare onset/associated**	42 (3)	544 (43)	496 (39)	308 (24)	911 (71)	401 (31.30)

Data from a public healthcare system including inpatient and outpatient services and a skilled nursing facility; NIT: nitrofurantoin, T/S: trimethoprim/sulfamethoxazole, CIP: ciprofloxacin, ESBL: extended-spectrum beta-lactamase, AMR: antimicrobial resistance, MDR: multidrug resistance. Not all ESBL-*E. coli* samples were tested for NIT, T/S or CIP

^*^7751 isolates tested for ESBL, Any AMR, or MDR, 7737 for T/S and CIP, 7736 tested for NIT

^**^1277 isolates tested for ESBL, Any AMR, or MDR, 1274 for T/S and CIP, 1273 for NIT

**Table 3:** Prevalence of antimicrobial resistance in healthcare-onset/associated *E. coli* bacteriuria episodes compared to those that are community-onset, 2014–2020

	RR (95% CI)					
	NIT	T/S	CIP	ESBL	Any AMR	MDR
Community onset (reference)						
Healthcare onset/associated	1.98 (1.40, 2.79)	1.23 (1.14, 1.32)	1.96 (1.80, 2.13)	3.00 (2.65, 3.39)	1.17 (1.13, 1.22)	1.86 (1.69, 2.05)

Data from a public healthcare system including inpatient and outpatient services and a skilled nursing facility; RR: risk ratio, NIT: nitrofurantoin, T/S: trimethoprim/sulfamethoxazole, CIP: ciprofloxacin, ESBL: extended-spectrum beta-lactamase, AMR: antimicrobial resistance, MDR: multidrug resistance

### Trend over time of bacteriuria episodes caused by drug-resistant E. coli

**Fig. 1:**
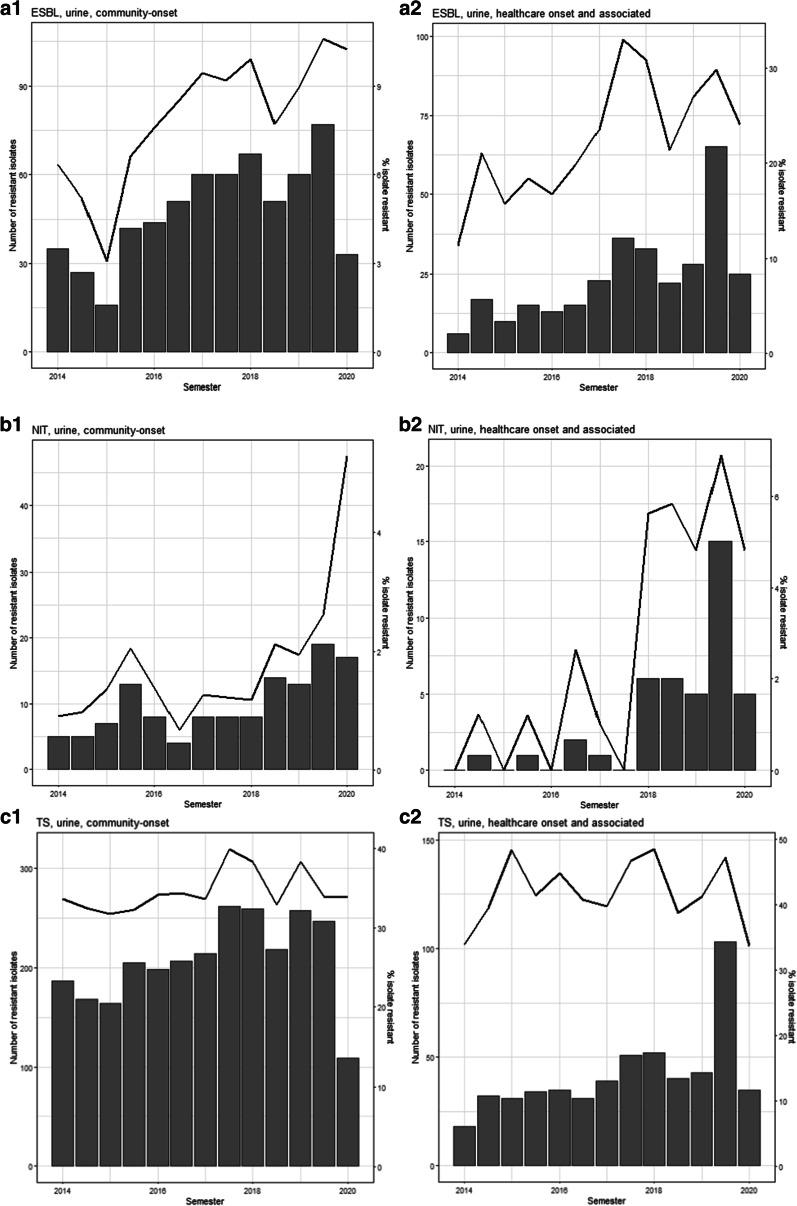

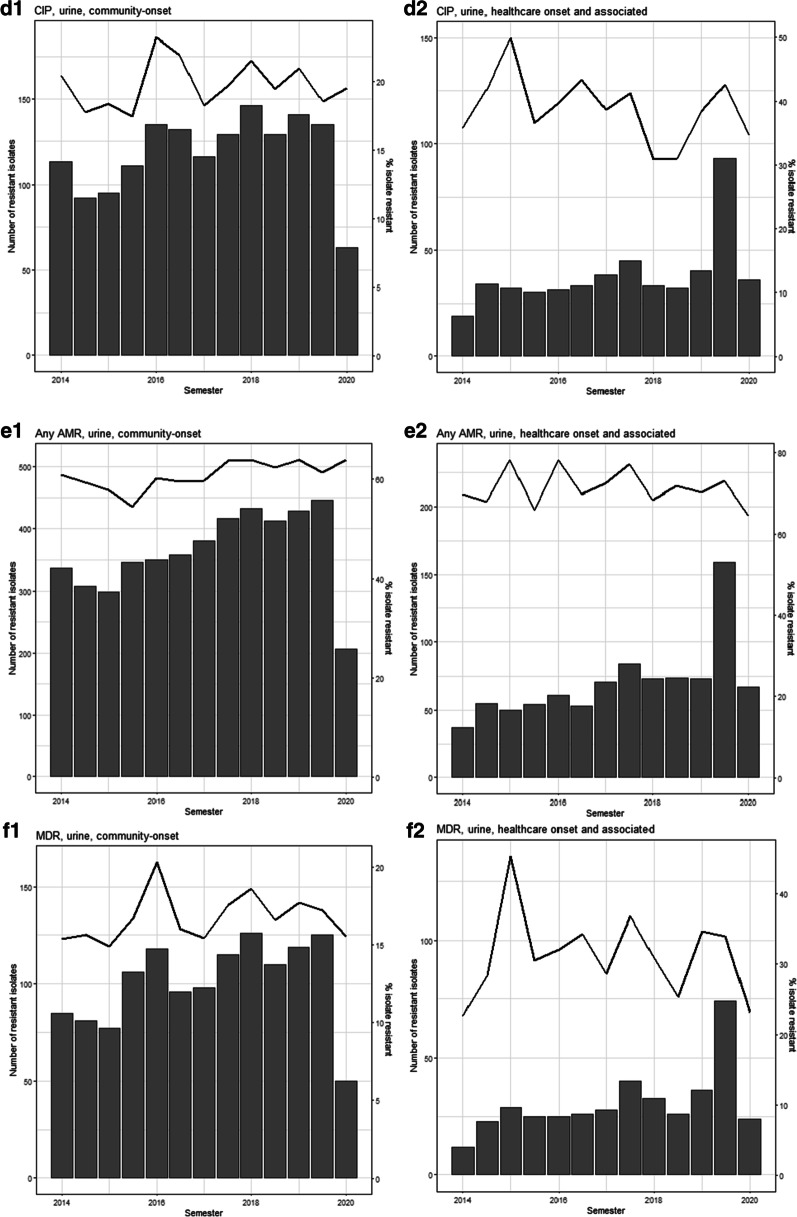
Temporal trend in community-onset vs healthcare-onset/associated bacteriuria caused by antimicrobial resistant *E. coli* by semester, 2014–2020. Note: bar = number of bacteriuria episodes, line = percent resistance. **a** ESBL-producing. 1. Community-onset. 2. Healthcare-onset/associated. **b** Nitrofurantoin. 1. Community-onset. 2. Healthcare-onset/associated. **c** Trimethoprim/sulfamethoxazole. 1. Community-onset. 2. Healthcare-onset/associated. **d** Ciprofloxacin. 1. Community-onset. 2. Healthcare-onset/associated. **e** Any resistance. 1. Community-onset. 2. Healthcare-onset/associated. **f** Multidrug resistance. 1. Community-onset. 2. Healthcare-onset/associated

ESBL-*E. coli* frequency in community-onset bacteriuria episodes increased from 6% in 2014 to 10% in 2020 (although 2020 included only 3 months of data), ranging from 5 to 10% and increasing an average of 0.91% (95% CI 0.56%, 1.26%) per year (Table 4 and Fig. 1). ESBL-*E. coli* frequency in healthcare-onset/associated bacteriuria episodes increased from 17% in 2014 to 24% in 2020, ranging from 17 to 29% and increasing an average of 2.31% (95% CI 1.01%, 3.62%) per year. ESBL-*E. coli* frequency increased an average of 1.03% (95% CI 0.67, 1.40) per year in outpatients and an average of 3.51% (95% CI 1.50, 5.52) per year in skilled nursing facility residents; it did not increase significantly in inpatients (Additional file 1: Table S2). Nitrofurantoin and trimethoprim-sulfamethoxazole resistance and resistance to any antimicrobial agent also increased (Table 4 and Fig. 1).

**Table 4:** Annual percentage point changes in prevalence of antimicrobial resistant *E. coli* from bacteriuria episodes, 2014–2020

	Annual percent point change (95% CI)					
	NIT	T/S	CIP	ESBL	Any AMR	MDR
Community onset	0.34 (0.16, 0.51)	0.64 (0.04, 1.23)	0.13 (− 0.37, 0.62)	0.91 (0.56, 1.26)	1.00 (0.40, 1.61)	0.25 (− 0.21, 0.72)
Healthcare onset/associated	1.61 (0.74, 2.47)	0.41 (− 1.07, 1.90)	− 0.59 (− 2.05, 0.87)	2.31 (1.01, 3.62)	− 0.18 (− 1.54, 1.18)	− 0.13 (− 1.52, 1.27)

Data from a public healthcare system; CI: confidence interval, NIT: nitrofurantoin, T/S: trimethoprim/sulfamethoxazole, CIP: ciprofloxacin, ESBL: extended-spectrum beta-lactamase, AMR: antimicrobial resistance, MDR: multidrug resistance. Logistic regression are univariate analyses including presence or absence of antimicrobial resistance to antibiotic and year from baseline

### Association between patient demographic characteristics and ESBL-*E. coli* bacteriuria by community-onset and healthcare-onset/associated bacteriuria

In univariate logistic regression models, among all *E. coli* bacteriuria episodes, community-onset ESBL-*E. coli* was associated with age over 65 years (risk ratio [RR] 1.93, 95% CI 1.26, 2.94), male gender (RR 2.24, CI 1.87, 2.69), and Chinese dialect (RR 1.37, CI 1.03, 1.81) or Spanish (RR 1.25, CI 1.05, 1.48) as a preferred language (Table 5). In models comparing ESBL-*E. coli* bacteriuria episodes to episodes caused by drug-resistant *E. coli*, older age, being male, and Chinese dialect as a preferred language, but not Spanish, were significantly associated.

**Table 5:** Association between patient demographic characteristics and ESBL-*E. coli* bacteriuria episode among all *E. coli* bacteriuria episodes or drug-resistant *E. coli* bacteriuria episodes, univariate analyses

	RR (95% CI)			
	Community-onset		Healthcare-onset/associated	
	ESBL-*E. coli* among all *E. coli* episodes	ESBL-*E. coli* among AMR *E. coli* episodes	ESBL-*E. coli* among all *E. coli* episodes	ESBL-*E. coli* among AMR *E. coli* episodes
**Age category (years)**				
0–17 (reference)				
18–34	0.76 (0.48, 1.19)	0.78 (0.50, 1.22)	1.88 (0.25, 14.08)	1.50 (0.20, 11.24)
35–64	1.44 (0.95, 2.18)	1.33 (0.87, 2.01)	3.97 (0.56, 28.33)	3.23 (0.45, 23.05)
65 +	1.93 (1.26, 2.94)	1.70 (1.12, 2.60)	2.66 (0.37, 19.07)	2.46 (0.34, 17.65)
**Gender**				
Female (reference)				
Male	2.24 (1.87, 2.69)	2.01 (1.67, 2.41)	1.61 (1.29, 2.02)	1.44 (1.50, 1.81)
**Race/ethnicity**				
Asian	1.07 (0.81, 1.41)	1.10 (0.83, 1.45)	0.74 (0.51, 1.07)	0.84 (0.58, 1.22)
Black	0.75 (0.54, 1.05)	0.80 (0.58, 1.12)	0.83 (0.59, 1.18)	0.77 (0.54, 1.09)
Latinx	1.23 (0.98, 1.55)	1.13 (0.89, 1.42)	1.10 (0.83, 1.46)	0.95 (0.72, 1.26)
Other	1.13 (0.79, 1.61)	1.09 (0.77, 1.56)	0.88 (0.53, 1.46)	0.84 (0.51, 1.40)
White (reference)				
**Preferred language**				
Chinese dialect	1.37 (1.03, 1.81)	1.36 (1.03, 1.81)	0.76 (0.48, 1.19)	0.89 (0.57, 1.39)
English (reference)				
Other	1.21 (0.81, 1.79)	1.23 (0.83, 1.82)	1.10 (0.63, 1.92)	1.13 (0.65, 1.98)
Spanish	1.25 (1.05, 1.48)	1.08 (0.91, 1.29)	1.01 (0.76, 1.31)	0.90 (0.67, 1.20)

Note: Univariate logistic regressions. Data from a public healthcare system; RR: risk ratio, ESBL: extended-spectrum beta-lactamase, AMR: antimicrobial resistant

In multivariate logistic regression models adjusted for age category, gender, race/ethnicity and preferred language, community-onset ESBL-*E. coli* bacteriuria was associated with age older than 65 years (RR 1.88, CI 1.17, 3.05) and male gender (RR 2.12, CI 1.65, 2.73), among all *E. coli* bacteriuria episodes (Table 6). Among bacteriuria episodes caused by drug-resistant *E. coli*, ESBL- *E. coli* bacteriuria was associated with male gender (RR 1.90, CI 1.50, 2.41) as well as age older 65 years (RR 1.60, CI 0.98, 2.61) although the CI included the null. Association of ESBL- *E. coli* bacteriuria with Latinx race/ethnicity was also observed although the CI included the null among all *E. coli* bacteriuria episodes (RR 1.52, CI 0.99, 2.33) and among bacteriuria episodes caused by drug-resistant *E. coli* (RR 1.39, CI 0.98, 1.98).

**Table 6:** Association between patient demographic characteristics and ESBL-*E. coli* bacteriuria episode among all *E. coli* bacteriuria episodes or drug-resistant *E. coli* bacteriuria episodes, multivariate analyses

	RR (95% CI)			
	Community-onset		Healthcare-onset/associated	
	ESBL-*E. coli* among all *E. coli* episodes	ESBL-*E. coli* among AMR *E. coli* episodes	ESBL-*E. coli* among all *E. coli* episodes	ESBL-*E. coli* among AMR *E. coli* episodes
**Age category (years)**				
0–17 (reference)				
18–34	0.84 (0.50, 1.41)	0.83 (0.51, 1.35)	1.87 (3.03E−19, 1.15E+19)	1.37 (3.78E−16, 5.01E+15)
35–64	1.41 (0.88, 2.26)	1.29 (0.81, 2.05)	3.92 (8.69E−23, 1.76E+23)	2.90 (4.64E−18, 1.81E+15)
65 +	1.88 (1.17, 3.05)	1.60 (0.98, 2.61)	2.74 (1.99E−22, 3.78E+22)	2.26 (4.17E−18, 1.23E+18)
**Gender**				
Female (reference)				
Male	2.12 (1.65, 2.73)	1.90 (1.50, 2.41)	1.53 (1.03, 2.26)	1.36 (0.96, 1.92)
**Race/ethnicity**				
Asian	0.96 (0.62, 1.48)	0.94 (0.62, 1.44)	0.78 (0.45, 1.35)	0.88 (0.51, 1.52)
Black	0.83 (0.52, 1.32)	0.83 (0.54, 1.26)	0.85 (0.50, 1.44)	0.79 (0.50, 1.26)
Latinx	1.52 (0.99, 2.33)	1.39 (0.98, 1.98)	1.35 (0.72, 2.54)	1.20 (0.73, 1.97)
Other	1.41 (0.84, 2.35)	1.28 (0.82, 1.99)	0.93 (0.50, 1.73)	0.94 (0.53, 1.66)
White (reference)				
**Preferred language**				
Chinese dialect	1.36 (0.86, 2.15)	1.39 (0.88, 2.21)	1.15 (0.53, 2.47)	1.12 (0.55, 2.25)
English (reference)				
Other	1.18 (0.76, 1.83)	1.25 (0.80, 1.94)	1.41 (0.77, 2.56)	1.33 (0.74, 2.39)
Spanish	0.97 (0.73, 1.30)	0.88 (0.67, 1.17)	0.90 (0.50, 1.60)	0.85 (0.53, 1.37)

Note: Multivariate logistic regressions including all variables presented above. Cluster bootstrap confidence intervals presented. Data from a public healthcare system; RR: risk ratio, ESBL: extended-spectrum beta-lactamase, AMR: antimicrobial resistant

For healthcare-onset/associated bacteriuria episodes, among all *E. coli* bacteriuria episodes, ESBL-*E. coli* was associated only with male gender in both univariate (RR 1.61, CI 1.29, 2.02) and multivariate (RR 1.53, CI 1.03, 2.26) analyses. Among episodes caused by drug-resistant *E. coli*, ESBL-*E. coli* bacteriuria was associated with male gender only in univariate analyses (RR 1.44, CI 1.50, 1.81).

The results of analysis of association between patient demographic characteristics and other antimicrobial agents are described Supplemental Tables 3 and 4.

### Association between patient demographic characteristics and ESBL-E. coli bacteriuria episodes by place of care

In multivariate regression models adjusted for age category, gender, race/ethnicity, preferred language, and hospitalization in the 90 days prior to urine culture, among all *E. coli* bacteriuria episodes from inpatients, male gender (RR 1.66, CI 1.13, 2.43) and hospitalization in the 90 days prior to urine culture (RR 2.35, CI 1.72, 3.20) were significantly associated with ESBL-*E. coli* bacteriuria (Additional file 1: Tables S5 and S6). Among bacteriuria episodes caused by drug-resistant *E. coli* in inpatients, male gender (RR 1.38, CI 0.98, 1.93), although the CI included the null, and hospitalization in the 90 days prior to urine culture (RR 1.97, CI 1.51, 2.57) were ESBL-*E. coli* bacteriuria.

For outpatients, in multivariate regression models, among all *E. coli* bacteriuria episodes, significant predictors of ESBL*-E. coli* bacteriuria were age older than 65 years (RR 1.62, CI 1.00, 2.62), male gender (RR 2.00, CI 1.52, 2.64), Latinx race/ethnicity (RR 1.65, CI 1.04, 2.62), and hospitalization in the 90 days before culture (RR 2.81, CI 2.04, 3.88). Among episodes caused by drug-resistant *E. coli*, ESBL*-E. coli* bacteriuria was associated with male gender and hospitalization in the 90 days before culture; association with Latinx race/ethnicity was noted, although the CI included the null (RR 1.47, CI 0.98, 2.22). No associations were found among skilled nursing facility residents (Additional file 1: Tables S5 and S6).

Results of associations between patient demographic characteristics and other antimicrobial agents are described in Additional file 1: Tables S5 and S6.

## Discussion

In this study of drug-resistant *E. coli* bacteriuria spanning more than 6 years in a safety-net public healthcare system serving a diverse population, we found antimicrobial resistant *E. coli* frequency to increase over time in both community and healthcare settings. The magnitude of the increase was greatest among ESBL-*E. coli* bacteriuria, which doubled in community settings and increased by more than 40% in healthcare settings. Older age, male sex, and Chinese dialect or Spanish as a preferred language (or, in some models, Latinx race/ethnicity) were associated with higher prevalence of ESBL-*E. coli* among all *E. coli* bacteriuria episodes.

Increasing prevalence of ESBL-*E. coli* in community-onset and healthcare onset/associated infections is now observed worldwide [4, 10, 12, 18–22]. A 2019 CDC report showed a 50% increase in hospital- and community-onset infections caused by ESBL-producing Enterobacteriaceae between 2012 and 2017 in the US [2]. A report from the Study for Monitoring Antimicrobial Resistance Trends (SMART) found prevalence of urinary tract infections caused by ESBL-*E. coli* to increase from 7.8 to 18.3% between 2010 and 2014 in the US, particularly among hospital-associated infections [23]. In contrast, the authors found increasing prevalence in community-onset infections in Canada [23]. Most recently, a report on urinary tract infections in US hospitalized patients found a prevalence of 17.2% for ESBL-producing Enterobacteriaceae [24]. We have previously shown increase in ESBL-*E. coli* bacteriuria cases in the same San Francisco public healthcare system, but were unable to decipher whether this increase occurred in community or healthcare settings [15]. Here, while prevalence and increase per year was greater in healthcare onset/associated ESBL-*E. coli* bacteriuria, especially in skilled nursing facility residents as shown in separate analyses, we also found a significant increase among community-onset bacteriuria.

We first compared ESBL-*E. coli* bacteriuria to bacteriuria caused by all other *E. coli* strains (non-ESBL drug-resistant and drug-susceptible *E. coli*), which would not necessarily distinguish risk factors associated with ESBL-*E. coli* from those associated with drug-resistant *E. coli*. Therefore, we performed secondary analyses comparing ESBL-*E. coli* bacteriuria to bacteriuria caused by non-ESBL drug-resistant *E. coli*. For community-onset ESBL-*E. coli* bacteriuria, we found older age and male gender to be associated risk factors. For healthcare-onset/associated bacteriuria, we found male gender to be associated with ESBL-*E. coli*.

The association with older age and male gender may represent complicated urinary tract infections more likely to occur in these populations, which may include catheter-associated infections or prostatitis requiring prolonged treatment with extended-spectrum beta-lactam drugs [25]. Since multidrug resistance is associated with ESBL-*E. coli*, factors contributing to frequent antibiotic exposures among older persons in community settings, such as frequent contact with healthcare, higher likelihood of recurrent urinary tract infection, and urinary retention requiring catheterization, may also contribute to the ESBL-*E. coli* selection [9, 11, 26–28].

Few studies have found differences in ESBL-*E. coli* infection by race/ethnicity, independent of healthcare exposures. A New York study found that children identified as Asian had greater odds of infection with ESBL-producing Enterobacteriaceae [13]. Studies utilizing genotyping methods have found that the majority of community-onset urinary tract infection caused by ESBL-*E. coli* are caused by major pandemic *E. coli* lineages belonging to specific sequence types, including ST131 and ST69 [9, 10, 29]. This may point to common-source exposures in the community. There is mounting evidence that infection with ESBL-*E. coli* is associated with international travel, particularly to South Asian countries, and food habits, including eating meat contaminated with ESBL-*E. coli* [11–14]. 

No study to our knowledge has found higher risk of ESBL-*E. coli* in Latinx populations. Our findings may represent increased access to antibiotics by this population in San Francisco, but prior studies from other regions in the US found no difference in access to and use of non-prescribed antibiotics among Latinx compared to non-Latinx individuals [30]. A majority of Latinx patients in this public healthcare system come from Mexico. Travel to Mexico may be a risk factor in our study population. A report from the SMART study showed that Mexico has the highest prevalence of community infections caused by ESBL-*E. coli* in Latin America [31]. Thus, unmeasured risk factors, such as travel and food consumption, may also be driving increasing community-onset bacteriuria caused by ESBL-*E. coli*. Additionally, we found that representation of Latinx patients in those with *E. coli* bacteriuria increased annually. While Covered California was founded in 2012, Healthy San Francisco, a program designed to provide healthcare services for uninsured San Francisco residents which works directly with our healthcare system, started in 2007. It is less likely that the increase in Latinx patients with *E. coli* bacteriuria is due to increased healthcare coverage, but perhaps to a change in use of healthcare services. The temporal increase and association of ESBL-*E. coli* bacteriuria among Latinx patients we observed in this study may have been driven by the temporal increase in this population group at risk.

While co-resistance of ESBL-*E. coli* to other antimicrobial agents, specifically fluoroquinolones and trimethoprim-sulfamethoxazole, is very common [3, 9, 32–34], even more concerning is our finding of phenotypic carbapenem co-resistance amongst ESBL-*E. coli*. We found that 12 (86%) of 14 carbapenem-resistant *E. coli* were ESBL-*E. coli*, although we do not have genetic information to evaluate whether they were carbapenemase-producers. A new report from the CRACKLE2 study found that 20% of non-carbapenemase-producing carbapenem-resistant Enterobacteriales isolated from hospitalized patients produced CTX-M, a common ESBL type [35]. Additionally, prior studies have found ESBL-*E. coli* co-resistance to nitrofurantoin to be either absent or negligible [36, 37]. Our finding that ESBL-*E. coli* co-resistance to nitrofurantoin was higher than that of co-resistance to trimethoprim-sulfamethoxazole may be representative of local antimicrobial resistance patterns, possibly driven by predominant *E. coli* sequence types.

There are several limitations to our study. First, community-onset cases were defined as cases with no history of hospitalizations in the 90 days at SFGH or LHH prior to urine culture. We did not obtain patient information before 90 days, when such patients could have had other healthcare exposures. Second, it may be that we underestimated hospitalization in the 90 days prior to urine culture if individuals were hospitalized in other healthcare systems. Prior studies, however, have shown high retention rate of patients within our public healthcare system [38]. Third, we combined community-onset healthcare-associated bacteriuria episodes with healthcare-onset ones. This is a simplified definition of healthcare-associated infections. Fourth, this is a retrospective study based on electronic health records, and thus we were not able to conduct molecular characterization of *E. coli* isolates. Lastly, while our study population is diverse in its racial/ethnic representation and their San Francisco neighborhoods, it is homogenous in that individuals receiving care in this public healthcare system have similar socio-economic circumstances. Thus, findings from our study may not be generalizable to other populations.

## Conclusion

Our findings raise concerning trends in both community and healthcare settings of ESBL-*E. coli* bacteriuria among patients examined at a San Francisco safety-net public health system. As bacteriuria, in particular urinary tract infections, often precede complications such as bacteremia and sepsis, this observation has serious implications for the clinical management of many types of infections. These findings also have important public health implications, emphasizing an increasing need for better surveillance and antibiotic stewardship programs for community-onset infections.

### Supplementary Information

**Additional file 1.** Association between patient demographic characteristics and antimicrobial-resistant E. coli by place of care (univariate and multivariate analyses) and number of bacteriuria episodes per year.

## Data Availability

The datasets used and/or analyzed during the current study are available from the corresponding author on reasonable request.

## Appendix

See Table

**Table 7:** Classes of antimicrobial agents reported

Antimicrobial class	Antimicrobial agents
Aminoglycosides	Amikacin
	Gentamicin
	Streptomycin
	Tobramycin
Carbapenems	Ertapenem
	Imipenem
	Meropenem
Cephalosporins	Cefazolin
	Cephalexin
	Cefuroxime
	Ceftazidime
	Ceftriaxone
	Cefotaxime
	Cefepime
	Ceftaroline
Macrolides	Azithromycin
	Clarithromycin
	Erythromycin
Penicillins	Penicillin
	Ampicillin
	Nafcillin-oxacillin
Beta-lactamase inhibitor combinations	Amoxacillin-clavulanate
	Ampicillin-sulbactam
	Piperacillin-tazobactam
	Ceftolozane-tazobactam
Quinolones	Ciprofloxacin
	Levofloxacin
	Moxifloxacin
Tetracyclines	Doxycycline
	Tetracycline
Glycopeptides	Vancomycin
Oxazolidinones	Linezolid
Rifamycins	Rifampin
Sulfonamides combination	Trimethoprim-sulfamethoxazole
Monobactam	Aztreonam
Lincosamides	Clindamycin
Lipopeptides	Daptomycin
Nitrofuran derivate	Nitrofurantoin
Nitro-imidazole derivatives	Metronidazole
Phosphonic	Fosfomycin
Amphenicols	Chloramphenicol

7.
